# Clinical Utility of Functional Near-Infrared Spectroscopy for Assessment and Prediction of Suicidality: A Systematic Review

**DOI:** 10.3389/fpsyt.2021.716276

**Published:** 2021-10-01

**Authors:** Y. Q. Lee, Gabrielle W. N. Tay, Cyrus S. H. Ho

**Affiliations:** ^1^Department of Psychological Medicine, Yong Loo Lin School of Medicine, National University of Singapore, Singapore, Singapore; ^2^Department of Psychological Medicine, National University Health System, Singapore, Singapore

**Keywords:** assessment, prediction, functional near-infrared spectroscopy, suicid*, major depressive disorder, schizophrenia, bipolar disorder, systematic review

## Abstract

**Introduction:** Suicide is a pressing psychiatric concern worldwide with no established biomarker. While there is some evidence of the clinical utility of functional near-infrared spectroscopy (fNIRS) in assessing and predicting suicidality, no systematic review of such evidence has been conducted to date. Therefore, this review aimed to systematically review and gather evidence from existing studies that used fNIRS signals to assess suicidality and its associated changes in the brain, and those that examined how such signals correlated with suicide symptomatology.

**Methods:** PubMed, EMBASE, and Cochrane Library databases were used in a systematic literature search for English-language articles published between 2000 and December 19, 2020 that focused on the utility of fNIRS for (i) assessing suicidality and its associated changes in the brain, and (ii) correlating with suicide symptomatology. Studies were included if they utilised fNIRS to evaluate variations in fNIRS-measured cerebral hemodynamic responses in patients with different mental disorders (e.g., major depressive disorder, schizophrenia), as well as in healthy controls, of any age group. Quality of evidence was assessed using the Newcastle-Ottawa quality assessment scale.

**Results:** A total of 7 cross-sectional studies were included in this review, all of which had acceptable quality. Across all studies, fNIRS demonstrated reduced cerebral hemodynamic changes in suicidal individuals when compared to non-suicidal individuals. One study also demonstrated the potential of fNIRS signals in correlating with the severity of suicidality.

**Conclusions:** This review provides a comprehensive, updated review of evidence supporting the clinical utility of fNIRS in the assessment and prediction of suicidality. Further studies involving larger sample sizes, standardised methodology, and longitudinal follow-ups are needed.

## Introduction

Suicide, which occurs across the lifespan, is a major public health concern, given that it remains an important contributor to mortality in all regions of the world (1). According to the World Health Organisation, suicide accounted for 1.4% of all deaths worldwide in 2016, and ~800,000 deaths every year are due to suicide. Suicidal ideation can be defined as thinking about or planning suicide, while a suicide attempt is a non-fatal, self-directed, potentially injurious behaviour with the intention to end one's life (2). For every adult death by suicide, it is estimated that there are more than 20 others who attempt suicide, with about 2.5% of the world's population attempting suicide at least once in their lifetime (3, 4). All things considered, there is evidently a pressing need for interventions, therapeutic or otherwise, aimed at reducing the risk of suicide and, consequently, preventing suicide more effectively (5, 6).

Despite the increasing global prevalence of suicide over the years, suicidality in individuals can still be said to be under detected, given its complex, multifaceted nature (7–9). Furthermore, reliable biological predictors for the risk of suicidality do not yet exist, given the almost exclusive reliance on self-reported suicide intent and the limited predictive value of identified non-biological risk factors and at present (10–12). As such, there exists a need for objective methods (i.e., those not biassed by opinion or interpretation) to ascertain suicidality in individuals swiftly so that appropriate intervention can be administered to ensure patient safety, especially since suicidal thoughts and behaviours have not declined appreciably in the past decades (10, 12). This can be done by assessing for, as well as making modifiable risk factors of suicide like trauma, pain, and social isolation, targets of clinical interventions (13–15). Traumatic experiences have shown to be an important risk factor for suicide, as they may be associated with significant psychosocial impairment (16). Additionally, a study by Serafini et al. (17) also found that an individual's unique sensory processing patterns, in addition to their traumatic experiences, are often involved in the pathophysiology of major affective disorders and other negative outcomes (e.g., suicidality). As such, they should also be considered when assessing for suicidality in the clinical setting.

In recent years, there has been a marked increase in the number of studies focusing on suicide-specific diagnosis and biomarkers, the latter of which are measurable biological parameters that increase risk of a disease, which includes brain imaging, genomic and biochemical markers (18–20). In particular, the use of brain-imaging modalities such as functional magnetic resonance imaging (fMRI), positron emission tomography (PET) and magnetoencephalography (MEG) has been employed to help in the diagnosis of psychiatric disorders and suicidality. fMRI, in particular, has also been used in the investigation of suicidal thoughts and behaviours (21). fMRI studies have provided evidence of increased suicide risk in association with differences in activation of areas of the brain, such as the prefrontal cortex (22). There are, however, limitations to the aforementioned imaging methods, including but not limited to high costs, various contraindications associated with the use of the imaging devices, and equipment inaccessibility.

In contrast, functional near-infrared spectroscopy (fNIRS) provides various advantages that makes it an ideal choice for interrogating brain function, one of which is that it is non-invasive (23). It also provides temporal resolution comparable to that of fMRI, involves neither ionising radiation nor loud noise, and is portable and relatively inexpensive, hence allowing for it to be repeated on patients as and when needed in their natural environment (24–27). Furthermore, it is readily amenable to integration with other technologies including electroencephalography (EEG) (28). fNIRS is a type of spectroscopy that utilises light sources between a spectral window of 650–1,000 nanometres, which can penetrate organic tissues and are preferentially absorbed by haemoglobin (29). Variations in oxygenated haemoglobin (oxy-Hb), used as an indicator of cortical activation, are then computed using the variance in absorbance using a modified version of the Beer-Lambert law (30). fNIRS research has been rapidly expanding across a wide range of areas in recent years (31). In psychiatry, fNIRS has been used in studies involving patients with major depressive disorder (MDD), schizophrenia and bipolar disorder (BD), in which such patients demonstrate considerably decreased prefrontal cortical activation in comparison to healthy controls when participating in cognitive tasks like the verbal fluency task (VFT) (32–34). The correlation between prefrontal cortical activation and suicidality has been less frequently examined and is, therefore, less established.

To the best of our knowledge, no systematic review has been conducted to date to evaluate the use of fNIRS in assessing and predicting suicidality. As such, the aim of this study was to systematically review and gather clinical evidence from the latest available literature, focusing on the utility of fNIRS for (i) assessing suicidality and its associated changes in the brain, and (ii) correlating with suicide symptomatology. Overall, we hope to provide a comprehensive, up-to-date review of the literature exploring the clinical utility of fNIRS as a biomarker for suicidality.

## Methods

### Data Sources and Search Strategy

We conducted this study using the Preferred Reporting Items for Systematic Reviews and Meta-Analysis (PRISMA) (35). A systematic review was completed based on English-language literature published between 2000 to 19 December 2020, focusing on the utility of fNIRS for (i) assessing suicidality and its associated changes in the brain, and (ii) correlating with suicide symptomatology. The three electronic databases utilised in the search were PubMed, EMBASE and Cochrane Library. The following search terms were used: “spectroscopy”, “near-infrared”, “near-infrared spectroscopy”, “fNIRS”, and “optical topography” (separated by OR), in combination with the term “suicid*”. These terms were searched as both text words and subject headings. Original research articles, human studies and conference proceedings in the English language were included, whilst abstracts, case reports and reviews were excluded. No funding was obtained for this systematic review.

### Eligibility Criteria and Data Collection

The primary outcome of interest for this systematic review was the variations in fNIRS-measured cerebral hemodynamic responses and their correlations with suicide symptomatology across patients with different mental disorders, as well as amongst healthy controls.

All studies retrieved from the databases were independently reviewed by two reviewers (YQL and CH) based on title and abstract. Where appropriate, full-text papers were extracted for a further inspection. Full-text reviews were subsequently conducted on selected studies that fulfilled the inclusion criteria, whereby the study characteristics and findings were extracted. Information included in data review consist of the authors, country, year published, conditions investigated, sample size and gender, mean age, diagnostic criteria, suicidality measure(s), medication use (if any), type of NIRS device utilised, paradigm utilised, brain areas studied, and main findings of the study.

### Quality Assessment of the Included Studies

As recommended by the Cochrane Collaboration (36), the Newcastle-Ottawa quality assessment scale (NOS) (37) was used to assess the risk of bias of the studies included in this review. The NOS assigns a maximum of 9 stars to each study with scores of 0–3, 4–6, and 7–9 indicating low, medium and high quality, respectively. These studies were independently rated by two reviewers (YQL and CH), and the results of this quality assessment are presented in Table 1. Any discrepancies in assessment were discussed before finalisation. All studies were deemed to be of acceptable quality, having been awarded a minimum of 4 stars.

**Table 1:** Risks of bias within studies selected.

**Selection**					**Comparability**	**Exposure**		
**Study sources**	**Is the case definition adequate?**	**Representative of cases**	**Selection of controls**	**Definitions of controls**		**Determination of exposure**	**Same method for determining cases and controls**	**Non-response rates**
Baik et al.	⋆		⋆	⋆	⋆	⋆		
Hirose et al.	⋆		⋆	⋆	⋆	⋆	⋆	
Tsujii et al.	⋆		⋆	⋆	⋆⋆	⋆	⋆	
Pu et al.	⋆		⋆	⋆	⋆	⋆	⋆	
Ota et al.	⋆		⋆	⋆	⋆	⋆	⋆	
Zahid et al.	⋆		⋆			⋆	⋆	
Matsuoka et al.	⋆		⋆	⋆	⋆	⋆	⋆	

*Each star represents one points (each study can obtain a maximum of 9 points), whereby the more stars given, the less prone to bias the study is*.

## Results

### Study Selection

**Figure 1:**
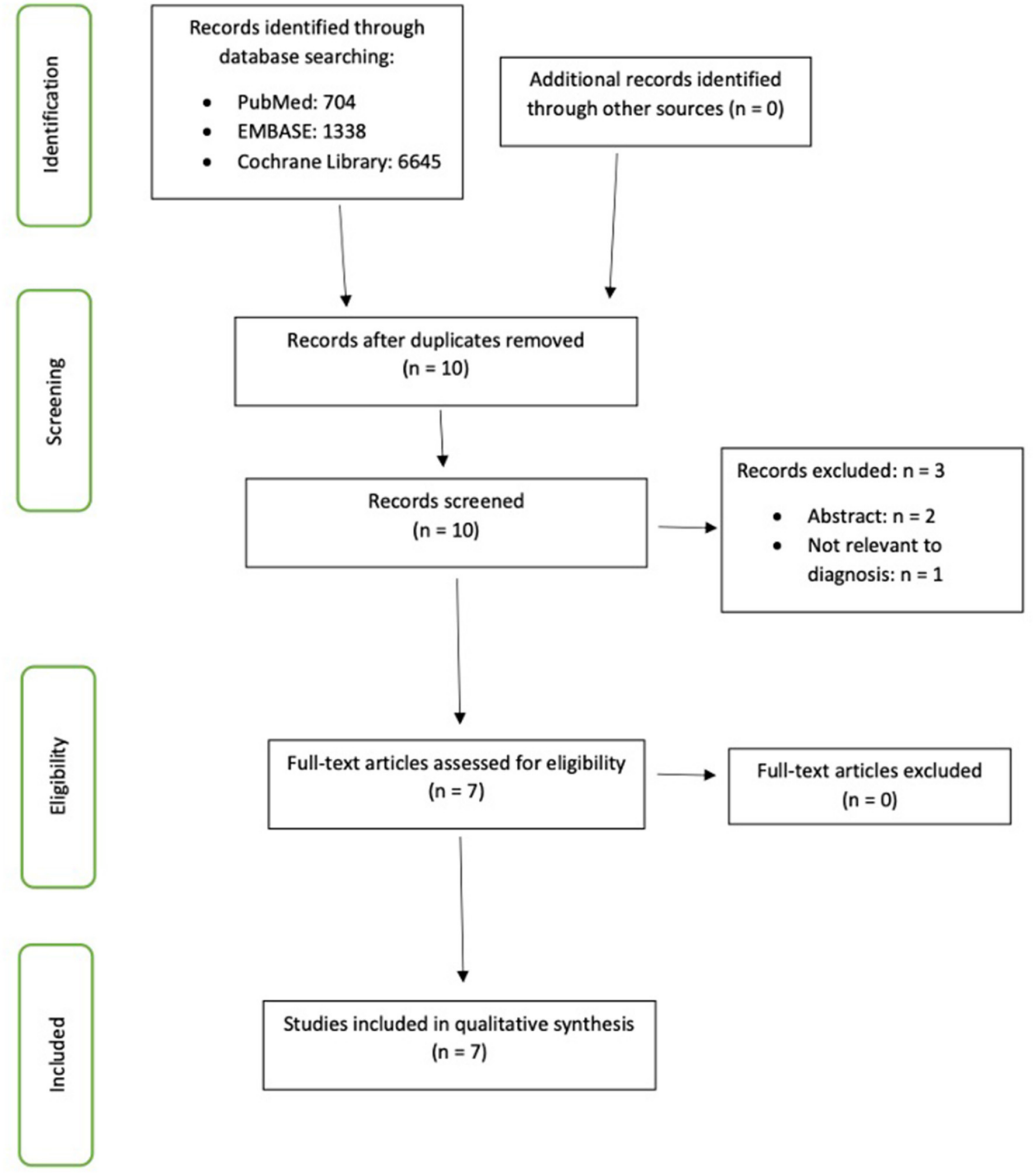
Preferred Reporting Items for Systematic Reviews and Meta-Analysis (PRISMA)-guided flow chart illustrating the screening and selection processes performed to identify the articles included in this review.

A total of 8,687 citations were identified from our database search, with 704 from PubMed, 1,338 from EMBASE, and 6,645 from Cochrane Library. After reviewing the titles, abstracts, and removing duplicated publications, 10 articles were selected. Of these, 7 studies met the inclusion criteria and were included in this analysis (Table 2). The selection process is displayed in Figure 1, constructed according to the PRISMA statement. Five of the studies were from Japan, one study from the USA and one study from Korea. All seven studies were cross-sectional in nature.

**Table 2:** Summary of peer-reviewed studies investigating functional near infrared spectroscopy (fNIRS)-measured cerebral hemodynamic responses and their correlation with suicidality.

**References**	**Country**	**Year**	**Condition**	**Sample size (male/female)**	**Age (mean ± standard deviation)**	**Diagnostic criteria (instrument)**	**Suicidality measure(s)**	**Medication**	**NIRS device**	**Paradigm**	**Brain areas**	**Main findings**
Baik et al. (38)	Korea	2019	MDD	MDD: 51 (17/34) HC: 63 (24/41)	MDD: 37.62 ± 14.36 HC: 33.42 ± 12.57	DSM-5	HAM-D	NIL	NIRSIT (OBELAB): 24 dual-wavelength laser diodes (780/850 nm) and 32 photo detectors separated by a 1.5 cm unit distance	VFT, Stroop task, two-back task	PF	- Relatively reduced left PF oxy-Hb changes for MDD patients - Positive correlation between VFT asymmetry index and HAM-D suicide item for MDD patients - Stronger effect of MDD severity on suicidal ideation with relatively greater association with left PF asymmetry for MDD patients
Hirose et al. (39)	Japan	2018	BD	BD, SA: 20 (6/14) BD, NA (control): 28 (14/14)	SA: 33.5 ± 11.4 NA: 38.7 ± 12.0	DSM-IV	History of suicide attempt; MINI	NIL	52-channel ETG-4000 (Hitachi)	VFT	F	- Smaller hemodynamic responses in various regions; delayed activation timing of NIRS signal in PF region for SA group - Significant, positive association between current suicide risk significantly and delayed activation timing in PF region for all BD patients
Tsujii et al. (40)	Japan	2017	MDD	MDD: 68 (24/44)- SA: 30 (8/22) - NA: 38 (16/22) HC: 40 (15/25)	MDD- SA: 37.6 ± 10.0 - NA: 38.8 ± 9.7 HC: 38.2 ± 10.5	DSM-4, HAM-D	History of suicide attempt	Daily doses converted to equivalent doses of Imipramine, chlorpromazine and diazepam	52-channel ETG-4000 (Hitachi)	VFT	PF, T	- Smaller hemodynamic response in left precentral gyrus for SA group - Significant correlation between severity of suicidal ideation and hemodynamic responses in right DLPFC for SA group
Pu et al. (41)	Japan	2015	MDD	MDD: 67 (29/38) HC: 67 (29/38)	MDD: 58.1 ± 16.0 HC: 58.1 ± 17.8	DSM-4	HAM-D	Daily doses of all antidepressants were converted to an equivalent dose of imipramine	52-channel ETG-4000 (Hitachi)	VFT	F	- Significantly smaller regional hemodynamic changes for MDD patients with suicidal ideation - Negative correlation between severity of suicidal ideation (as measured by HAM-D) and hemodynamic changes in DLPFC, OFC and FPC regions for MDD group
Ota et al. (42)	Japan	2020	ASD	ASD: 20 (16/4) HC: 29 (16/4)	ASD: 29.05 ± 6.39 HC: 27.20 ± 4.16	DSM-5	MINI	NIL	24-channel ETG-4000 (Hitachi)	VFT	PF	- Significant positive correlation between current suicide risk score and centroid value in PF region for ASD patients
Zahid et al. (43)	USA	2020	NIL	296 (67/229)	18.8 ± 1.2	NIL	BDI;	NIL	Model 200A (fNIR Devices LLC)	Anagram tasks	DLPFC	- DLPFC activity associated with suicidal ideation in sex-specific ways
Matsuoka et al. (44)	Japan	2020	SZ (ROSZ)	SZ: 86 (46/40)- SA: 24 (12/12) - NA: 62 (34/28) HC: 119 (68/51)	SA: 25.4 ± 6.6 NA: 25.9 ± 7.3 HC: 26.5 ± 5.1	DSM-4	History of suicide attempt	Daily doses of all AP, BZD and APK drugs were converted to chlorpromazine, diazepam and biperiden equivalent doses	52-channel ETG-4000 (Hitachi)	LFT	F	- Right DLPFC brain activity of SA subgroup significantly lower than NA group

*MDD, Major depressive disorder; HC, Healthy control; DSM, Diagnostic and Statistical Manual; HAM-D, Hamilton Rating Scale for Depression; VFT, Verbal fluency test; PF, Prefrontal; BD, Bipolar disorder; SA, Suicide attempter; NA, Non-suicide attempter; MINI, Mini International Neuropsychiatric Interview; F, Frontal; T, Temporal; DLPFC, Dorsolateral prefrontal cortex; OFC, Orbitofrontal cortex; FPC, Frontopolar cortex; ASD, Autism spectrum disorder; BD, Beck's Depression Inventory; SZ, Schizophrenia; ROSZ, Recent-onset schizophrenia; AP, Antipsychotic; BZD, Benzodiazepine; APK, Antiparkinsonian; LFT, Letter fluency task*.

### Correlation of fNIRS Signals With Suicidality

All 7 papers reported on fNIRS studies, in which changes in cerebral hemodynamic responses were correlated with suicidality in a total of 954 participants. These studies were inclusive of patients with MDD, schizophrenia, bipolar disorder and autism spectrum disorder (ASD), as well as healthy individuals. The majority of the studies adopted the VFT, except for one study that used anagram tasks, as their paradigm. The fNIRS instruments utilised included the 52-channel ETG-4000 (Hitachi), 24-channel ETG-4000 (Hitachi), NIRSIT (OBELAB), and Model 200A (fNIR Devices LLC).

According to our assessment of the author's conclusions, studies on MDD showed oxy-Hb concentration values have significant negative correlation with suicidality in the sample population based on measures such as the Hamilton Depression (HAM-D) scores as well as patient's previous history of suicide attempts. Tsujii et al. (40) also demonstrated that significant negative correlation was still present after correcting for confounding factors such as age, gender and estimated IQ, and the suicide attempt (SA) group was noted to have smaller hemodynamic response than the non-attempt (NA) and healthy control (HC) group in the left precentral gyrus during VFT. VFT also induced widespread frontotemporal cortical activation in HCs and NAs, whereas SAs showed significant activation only in the left superior frontal gyrus, left middle frontal gyrus, bilateral inferior frontal gyrus, and right precentral gyrus. SAs also showed significant correlation between the severity of suicide ideation and hemodynamic responses in the right dorsolateral prefrontal region, in contrast to NAs who did not show such correlation. Hemodynamic responses in the right middle frontal gyrus were also negatively correlated with aggression and hopelessness in the SAs but not in the HCs and NAs.

Such negative correlation was corroborated by Baik et al. (38), who also showed that MDD patients have relatively reduced left prefrontal oxy-Hb changes during VFT than HCs, and amongst the MDD patients, there was significant positive correlation between asymmetry index for VFT and the suicide item of HAM-D. With relatively greater left prefrontal asymmetry, the effect of depression severity on suicide ideation was also noted to be stronger. Additionally, Pu et al. (41) also noted that regional hemodynamic changes in the right dorsolateral prefrontal cortex (DLPFC), orbitofrontal cortex (OFC), and right frontopolar cortex (FPC) regions in patients with MDD with suicidal ideation were significantly smaller than those without suicidal ideation, and similar differences were also noted between patients with MDDs and HCs. In patients with MDD, hemodynamic changes during VFT correlated negatively with the severity of suicidal ideation (as measured by the HAM-D questionnaire), in the DLPFC, OFC, and FPC regions.

In patients with BD, similar findings were discovered. Hirose et al. (39) showed that depressed BD patients with history of SA had smaller hemodynamic responses by VFT in the bilateral precentral and superior temporal gyri, and left supramarginal, inferior frontal, post-central and middle temporal gyri, as compared to BD patients without history of suicide attempts. SA patients also exhibited delayed activation timing of the NIRS signal in the PF region, and delayed activation timing of NIRS signal was significantly and positively associated with current suicide risk.

Assessing prefrontal hemodynamic response and suicide risk in ASD, Ota et al. (42) showed that there was significant positive correlation between the current suicide risk score, and their centroid value in the prefrontal region, while there was no significant differences between the ASD and control groups in terms of the mean oxy-Hb changes induced by VFT for each channel.

Matsuoka et al. (44) assessed prefrontal dysfunction and history of suicide attempts among patients with recent onset schizophrenia, and results showed that SA group had significantly lower brain activity in the right DLPFC during the letter fluency task as compared to the NA group.

Finally, Zahid et al. (43) assessed 296 undergraduates for suicidal ideation and monitored them using a fNIRS device whilst they engaged in anagram tasks. This study found that DLPFC activity was associated with suicidal ideation in gender-specific ways. During the homogenous 3-letter anagram trials, males who reported suicidal ideation displayed significantly reduced levels of blood oxygenation across the DLPFC compared to males who did not suicidal ideation, which indicated reduced neural activation during the task. In contrast, females who did not report suicidal ideation displayed reduced levels of blood oxygenation compared to females who reported suicidal ideation. As for the 5-letter anagrams task during the event-related trials, male who reported suicidal ideation displayed elevated levels of blood oxygenation and neural activation at several locations compared to males who did not report suicidal ideation, while females who reported suicidal ideation tended to exhibit lower levels of neural activation than those who did not report suicidal ideation. The findings from this study suggested that there are distinct patterns of neural activity across the DLPFC that differentiate individuals who report suicidal ideation from those who do not, and the patterns are dependent on both the gender and nature of the cognitive task allocated.

## Discussion

Considering the findings of the reviewed papers, our systematic review demonstrates the clinical potential of fNIRS signals as a biomarker that differentiates suicidal individuals from non-suicidal individuals. A few studies also found that smaller prefrontal (PF) and DLPFC hemodynamic responses during cognitive tasks correlated with the severity of suicidal ideation. As a reliable, established biomarker for suicidality does not yet exist, such information would be fundamental in determining the extent to which fNIRS can be used to assess and predict suicidality in a clinical setting.

To date, this is the first systematic review on fNIRS findings in relation to suicidality with the goal to provide a comprehensive, up-to-date overview of information surrounding the utility of fNIRS for (i) assessing suicidality and its associated changes in the brain, and (ii) correlating with suicide symptomatology in different study populations (i.e., patients with different psychiatric disorders, as well as healthy controls). Based on the collective findings of the reviewed papers, most of the studies were conducted in Japan, while the most common paradigm used is the VFT, a common, validated neuropsychological test used to ascertain executive function and language content (45, 46). Participants of the VFT are typically instructed to generate as many unique words as possible that begin with a particular letter during the task for a restricted duration of time varying between 30 and 60 s (47). The VFT has shown to be able to elicit distinct differences between depressed patients and healthy control in both performance and neuroimaging responses (46, 48).

Conversely, all seven studies focused on hemodynamic changes in discrete brain regions. It would also be worthwhile to explore how functional connectivity, which is the temporal dependency of neuronal activation patterns of anatomically separated brain regions (49), differs between SAs, NAs, and HCs. Also, none of them demonstrated data on the sensitivity and specificity comparing suicidal and non-suicidal individuals, which would be important in determining the clinical validity of fNIRS as a biomarker for suicidality. This points to the need for future studies with standardised methods of analyses, which would then allow for more accurate comparisons to be made (50). In a similar vein, despite the use of the VFT as the paradigm in most of the studies reviewed, there were variations in other factors such as the devices used to measure NIRS signals or how suicidality was measured, which reduces the validity of the comparisons made. Future research focusing on the clinical utility of fNIRS in assessing and predicting suicidality could also include longitudinal studies with longer follow-up durations spanning 6–12 months. All the reviewed studies are cross-sectional in nature, meaning that they are unable to provide any information on the prognosticative potential of fNIRS for suicidality. Longitudinal studies would be beneficial in providing a better understanding of the fNIRS-measured cerebral hemodynamic responses as a marker for suicidality and investigate whether it is a state or trait-dependent marker of suicidality.

Despite the several advantages of fNIRS over other imaging modalities such as fMRI, its limitations cannot be discounted. First, near-infrared light has limited spatial resolution (around 1 cm) and depth of penetration, thus rendering it unsuitable in the measuring of cortical hemodynamic responses to cognitive stimuli when deep brain regions with crucial roles in psychiatric disorders are involved (25, 51). Studies involving the use of fMRI, on the other hand, found disrupted neural responses in both the insula cortex and subgenual anterior cingulate cortex in depressed SAs, but not in depressed NAs and HCs, which led to greater aversion to uncertainty (52, 53). Jung et al. (54) also found that depressed NAs had decreased functional neural network connectivity (FNC), and that the degree of FNC was associated with suicidal ideation. Second, layered and/or dark-coloured hair attenuate near-infrared light, resulting in poor optical contact and affecting signal quality (51, 55). Third, fNIRS signals are susceptible to corruption by signals arising from respiratory rate, heart rate and blood pressure fluctuations, thus affecting signal interpretation (33, 56). Taken together, however, the effects of these limitations on fNIRS data could be alleviated by statistical data processing and enhanced data-collecting protocols (24, 28, 30, 56). The use of other neuroimaging modalities like fMRI could also be employed to supplement the findings of fNIRS scans, thus allowing for a more comprehensive assessment of neurophysiological changes.

Our review also comes with certain limitations. First, at the time of our study, there was limited existing literature on the topic of fNIRS and its correlation with suicidality. As such, the data available formed a small sample size, leading to a higher risk of confounding and selective biases, along with reduced power. Furthermore, out of the seven studies reviewed, five were conducted in Japan, hence limiting the ecological validity of our findings. However, this review was able to synthesise the information available to provide an up-to-date overview of studies exploring fNIRS and its clinical utility in assessing and predicting suicidality, hence facilitating future research and developments in this topic. Second, most of the studies used the VFT as the paradigm. Although the VFT is simple and can be conventionally administered, it would be worthwhile to ascertain the effects of other paradigms such as the Emotional Stroop task and investigate other brain regions besides the prefrontal cortex. Third, we only included papers published in English and searched in three databases, and there may have been publication bias as there may have been other papers published in other languages and/or on other platforms. Nevertheless, our review's strength lies in it being the first systematic review to evaluate the use of fNIRS in assessing and predicting suicidality. It also provides a comprehensive overview of studies that have been conducted in this area of the fNIRS research landscape.

In conclusion, current literature has provided sufficient evidence regarding fNIRS as a complementary tool for the assessment and prediction of suicidality in the clinical setting, as there have been consistent attenuated hemodynamic signals in different cortical brain regions, along with correlations between hemodynamic changes and degree of suicidality, when comparisons are made across depressed patients with a history of suicide attempts, those with no history of suicide attempts, and healthy controls. As such, the result of an individual's fNIRS scan could potentially be taken as an objective measure of suicidality; it could also be indicative of one's predisposition to suicidality. While our systematic review has shed light on the promising potential of fNIRS to be used as an objective method of assessing and predicting suicidality, further methodological improvements, such as those aimed at enhancing signal quality, are of crucial importance in ensuring replicability in future studies. Future studies with larger sample sizes, standardised methodology, as well as longitudinal follow-up with participants, are also recommended to further our knowledge of fNIRS and its clinical utility in psychiatric clinical practise and research.

## Data Availability Statement

The original contributions presented in the study are included in the article/supplementary material, further inquiries can be directed to the corresponding author.

## Author Contributions

YL and CH: conceptualization, data extraction, and review. YL and GT: writing. CH: review and manuscript amendment. All authors contributed to the article and approved the submitted version.

## Conflict of Interest

The authors declare that the research was conducted in the absence of any commercial or financial relationships that could be construed as a potential conflict of interest.

## Publisher's Note

All claims expressed in this article are solely those of the authors and do not necessarily represent those of their affiliated organizations, or those of the publisher, the editors and the reviewers. Any product that may be evaluated in this article, or claim that may be made by its manufacturer, is not guaranteed or endorsed by the publisher.
